# A Baybayin word recognition system

**DOI:** 10.7717/peerj-cs.596

**Published:** 2021-06-16

**Authors:** Rodney Pino, Renier Mendoza, Rachelle Sambayan

**Affiliations:** Institute of Mathematics, University of the Philippines Diliman, Quezon City, Metro Manila, Philippines

**Keywords:** Baybayin, Optical character recognition, Support vector machine, Baybayin word recognition

## Abstract

Baybayin is a pre-Hispanic Philippine writing system used in Luzon island. With the effort in reintroducing the script, in 2018, the Committee on Basic Education and Culture of the Philippine Congress approved House Bill 1022 or the ”National Writing System Act,” which declares the Baybayin script as the Philippines’ national writing system. Since then, Baybayin OCR has become a field of research interest. Numerous works have proposed different techniques in recognizing Baybayin scripts. However, all those studies anchored on the classification and recognition at the character level. In this work, we propose an algorithm that provides the Latin transliteration of a Baybayin word in an image. The proposed system relies on a Baybayin character classifier generated using the Support Vector Machine (SVM). The method involves isolation of each Baybayin character, then classifying each character according to its equivalent syllable in Latin script, and finally concatenate each result to form the transliterated word. The system was tested using a novel dataset of Baybayin word images and achieved a competitive 97.9% recognition accuracy. Based on our review of the literature, this is the first work that recognizes Baybayin scripts at the word level. The proposed system can be used in automated transliterations of Baybayin texts transcribed in old books, tattoos, signage, graphic designs, and documents, among others.

## Introduction

Baybayin is a pre-colonial writing system primarily used by Tagalogs in the northern Philippines. Currently, Baybayin is an obsolete writing script but it has penetrated the interest as a design for a tattoo or for Filipino-themed apparel (Cabuay, 2009). In April 2018, the Committee on Basic Education and Culture of the Philippine Congress signed House Bill 1022 that states the national writing system of the Philippines is the Baybayin. Further, the said bill requires the local manufacturers to imprint Baybayin scripts with their translation on product labels, and at least four (4) Executive Departments are assigned to promulgate the said script (Lim & Manipon, 2019).

**Figure 1:**
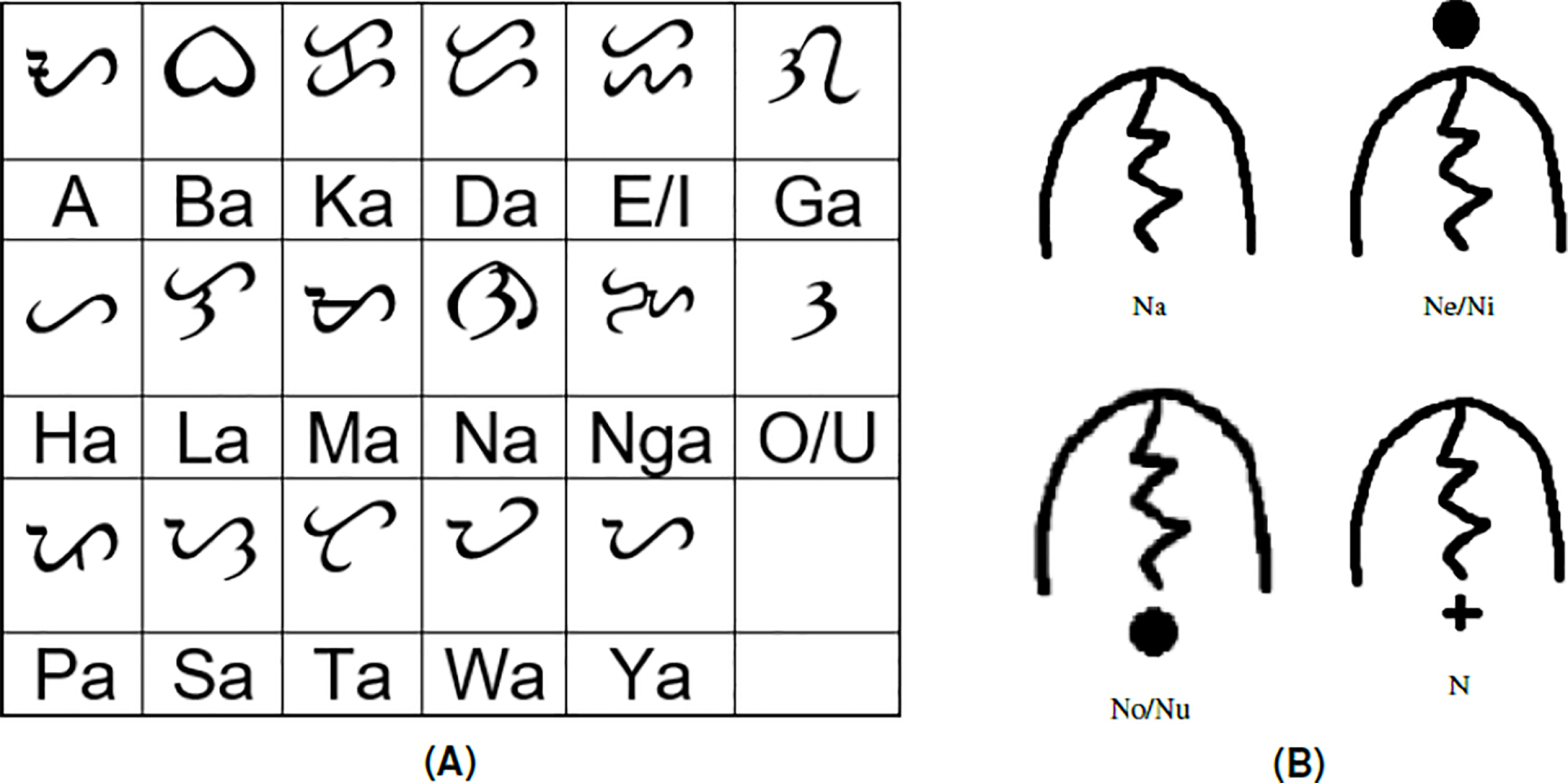
The Baybayin writing system: (A) the Baybayin alphabet (14 syllabic consonants and three vowels) and their Latin equivalent and (B) the placement of diacritics to indicate the different pronunciations of a consonant syllable.

The Baybayin is a left-to-right writing system of the Tagalog language. Its alphabet comprises 17 main characters, 14 of which are (syllabic) consonants, and the remaining three are vowels (see Fig. 1A). Each consonant character is read with a default vowel sound ‘ ∖a∖’. One can express the other vowels by employing diacritics or accents. For example, an accent written below a consonant character may represent an accompaniment vowel ‘ ∖o∖’ or ‘ ∖u∖’ sound. A diacritic placed above a consonant character may have pronounced vowels ‘ ∖e∖’ or ‘ ∖i∖’. Utilizing diacritics can also be interpreted to silence the vowel sounds. Figure 1B shows an instance of the distinguishable phonetic features of a Baybayin consonant character using diacritics.

The accent symbols used for the Baybayin script are bar, dot, and cross. With respect to their location, a dot or a bar represents the vowels E/I or O/U, while the cross symbol placed underneath the character silenced the vowel “a” (see Fig. 1B) (Cabuay, 2009). A sample of a Baybayin-written word and its Latin transliteration is shown in Table 1.

**Table 1:** A Baybayin word and its equivalent Latin conversion. *Matematika* is the Tagalog word for *Mathematics*.

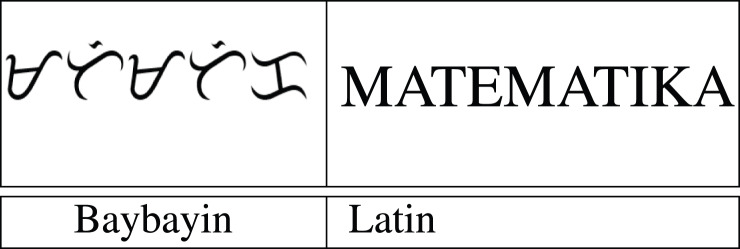
	

With recent advancements and innovations, machine learning is one of the most powerful technologies in today’s world. Every human that uses any technology has benefited from machine learning. Some of its countless applications can be found in security systems (Sagar, Jhaveri & Borrego, 2020; Panigrahi et al., 2021), biometric measurements (Chaurasia, Kohli & Garg, 2014), software developments (Chandra et al., 2016), and fraud news detection (Hakak et al., 2021). One contribution of machine learning that is a continuously developing field is optical character recognition (OCR). OCR is a technology that automatically recognizes characters through an optical mechanism. It is designed to process and read images that consist entirely of text, in handwritten or typewritten form Mithe, Indalkar & Divekar (2013). OCR research studies consider several, or a particular level for recognition: on-page, line, block, word, or character level (Ghosh, Dube & Shivaprasad, 2010).

Studies on Baybayin character recognition have started gaining popularity. The first Baybayin OCR study was done by Recario et al. (2011), where they have presented a system that reads automatically the Baybayin characters and outputs the equivalent Latin syllables. Their method utilized the freeman chain coding and line angle categorization for classification, where they obtained 66.47% and 51.96% recognition rates, respectively. Nogra, Romana & Maravillas (2019) and Nogra, Romana & Balakrishnan (2020) have reported Baybayin character recognition schemes that convert the input to a corresponding Latin syllable using Long Short-Term Memory (LSTM) neural network (2019) and Convolutional Neural Network (CNN) (2020), with 92.9% and 94% recognition accuracies, respectively. Daday, Fajardo & Medina (2020) have introduced the feed-forward neural network (FFNN) and CNN for Baybayin script classification. Both network models use a dropout method and have yielded 92.4% and 91.69% recognition rates, respectively. Bague et al. (2020) proposed a CNN model for Baybayin character recognition with a Visual Geometry Group 16 (VGG16 type network), where they calculated a 98.84% accuracy. These Baybayin OCR studies in the literature are based at the character level, indicating its early development. Recio & Mendoza (2019) employed a three-step detection approach to edges of texts images with Baybayin transcriptions.

In Pino, Mendoza & Sambayan (2021), a Baybayin character recognition system has been proposed using SVM, which is a classification algorithm with extensive applications in data categorization (Bishop, 2006). SVM has attracted researchers because of its robustness and high recognition accuracy (Thomé, 2012). Applications of SVM can be found in various fields of science and engineering (Thomé, 2012; Sapankevych & Sankar, 2009; Nayak, Naik & Behera, 2015; Yang, 2004; Rivero, Lemence & Kato, 2017; Rivero & Kato, 2018; Do & Le, 2019; Le et al., 2019; Le, 2019; Byun & Lee, 2003). The OCR system proposed by Pino, Mendoza & Sambayan (2021) consists of four SVM classification models, all of which have recognition rates above 96% accuracy.

Although several systems have been proposed for recognizing Baybayin characters, we believe that none has been formulated for reading Baybayin at the word level. This work aims to fill this research gap. Various machine learning algorithms have been used in word-level recognition of different writing systems. Using Gabor filters and four classifier systems, Jaeger, Ma & Doermann (2005) have reported a script identification system that discriminates Latin from Arabic, Korean, and Hindi writing systems. Their work yields a 97.39% recognition rate in categorizing Latin from Hindi script. With 97.06% average recognition rate, Hangarge, Santosh & Pardeshi (2013) have distinguished six Brahmic scripts, namely, Kannada, Devanagari, Tamil, Malayalam, Latin, and Telugu, using directional discrete cosine transforms and linear discriminant analysis. Arica & Yarman-Vural (2002) have proposed a scheme in recognizing cursive handwritten Latin scripts by using Hidden Markov Model (HMM) for classification and combined it with lexicon information, where they obtained a 92.3% recognition rate. An approach using an unsupervised feature learning algorithm and CNN for Latin scripts word-level recognition was presented by Wang et al. (2012) in which they acquired an 83.9% accuracy. For Arabic script, Erlandson, Trenkle & Vogt (1996) have proposed a word-level recognition by extracting morphological details of an Arabic word image and matching its feature vectors. The study has concluded with a 65% recognition accuracy. With 91.38% word recognition accuracy, Sankaran & Jawahar (2012) have proposed a recognition scheme for printed Devanagari script using bidirectional long short-term memory (Bi-LSTM). The pyramid histogram of oriented gradient feature with an SVM classifier was used to recognize Bangla script at word level as reported by Bhunia et al. (2015), where the recognition accuracy yields 97.23%. Pham & Le-Hong (2017) demonstrated a Vietnamese-named entity recognition where they utilized a combination of Bi-LSTM, CNN, and conditional random field (CRF) models. Their work resulted in an 88.59% *F*_1_ Score. A pragmatic mathematical approach has been proposed by Gao et al. (2005) for Chinese word recognition. Their result obtained an accuracy of 95.7% using a vector space model-inspired classifier. Using HMM, Dehghan et al. (2001) proposed a holistic word recognition technique for handwritten Arabic scripts, where they got a 65.05% recognition rate. Another word-based Arabic script recognition system had been reported by AlKhateeb et al. (2008), where they utilized a Discrete Cosine Transform (DCT) technique for feature extraction and multilayer perceptron (MLP) neural network for classification. The study achieved an 82.5% recognition accuracy. Kessentini, Paquet & Ben Hamadou (2010) have proposed an independent-script word recognition system on offline handwritten writing systems. They make use of multi-stream HMMs and implemented their method on Latin and Arabic scripts, where they yielded an 89.8% and 79.8% recognition performance, respectively. Ghosh, Roy & Kumar (2018) proposed an online handwritten word recognition for four major Indic scripts - Devanagari, Bengali, Telugu, and Tamil. The system uses two zone-wise features and an HMM-based classifier for the categorization process. They obtained an impressive 96.55%, 93.34%, 88.34%, and 93.47% recognition rates, respectively, for the considered scripts using 1000 lexicon size. Another study by Ghosh, Vamshi & Kumar (2019) utilized the horizontal zone features and RNN based models, LSTM and Bi-LSTM networks, to recognize non-cursive Devanagari and Bengali scripts. Their proposed method achieves a superior 99.50% and 95.24% recognition accuracies, respectively. A cross-language approach has been presented by Bhunia et al. (2018) to recognize at word level the three low resource Indic scripts, namely, Bangla, Devanagari, and Gurumukhi. HMM and SVM models were used to classify each zone level of a word, where they obtained a 75.21% word recognition accuracy. A comprehensive survey study on word OCR systems by Kaur & Kumar (2018) shows that the research area is still in development for Indic and non-Indic scripts and suggested more research studies need to be done.

The Baybayin word recognition algorithm proposed in this study relies heavily on the OCR system proposed in Pino, Mendoza & Sambayan (2021). For brevity, we will refer to this method as the SVM-OCR system. We segment a given Baybayin word into its character components and use the SVM-OCR system to identify the Latin syllable equivalent of each component. These Latin syllables are concatenated to form the equivalent word of the Baybayin word input. However, the formed Latin script might not be a Tagalog word because some syllables use the same character recognition. For example, Baybayin does not discriminate the vowel ’e’ from ’i’, which means, ’ne’ and ’ni’ are written in the same way (see Fig. 1B). Thus, one needs to check whether the constructed Latin script belongs to a Tagalog dictionary. The main contributions of this paper are as follows:

1. Compile novel datasets for Baybayin word images and Tagalog dictionary.
2. Use SVM to find the equivalent of a Baybayin word in Latin alphabet.
3. Determine all the other possible equivalent words by cross-checking the Tagalog dictionary.
4. Show that the proposed scheme has a high recognition accuracy when tested on the dataset of Baybayin word images.

The remainder of the paper proceeds as follows: ‘Dataset Collection’ discusses how Baybayin word images and Tagalog word dictionary are gathered and compiled. The proposed OCR algorithm for Baybayin word-level recognition is presented in ‘Proposed System’. In ‘Recognition Setup, Results and Discussions’, we present the results and discussion of our proposed system. We give our concluding remarks and recommendations in ‘Conclusions and Future Works’.

## Dataset Collection

This section presents the process on how we collect images of Baybayin words and compile a Tagalog dictionary. The collection of Baybayin word images will be used to assess the system’s performance. The formed Latin script will be checked if it is in the Tagalog dictionary. These datasets can be accessed publicly in Pino (2021a) and Pino (2021b), respectively.

**Figure 2:**
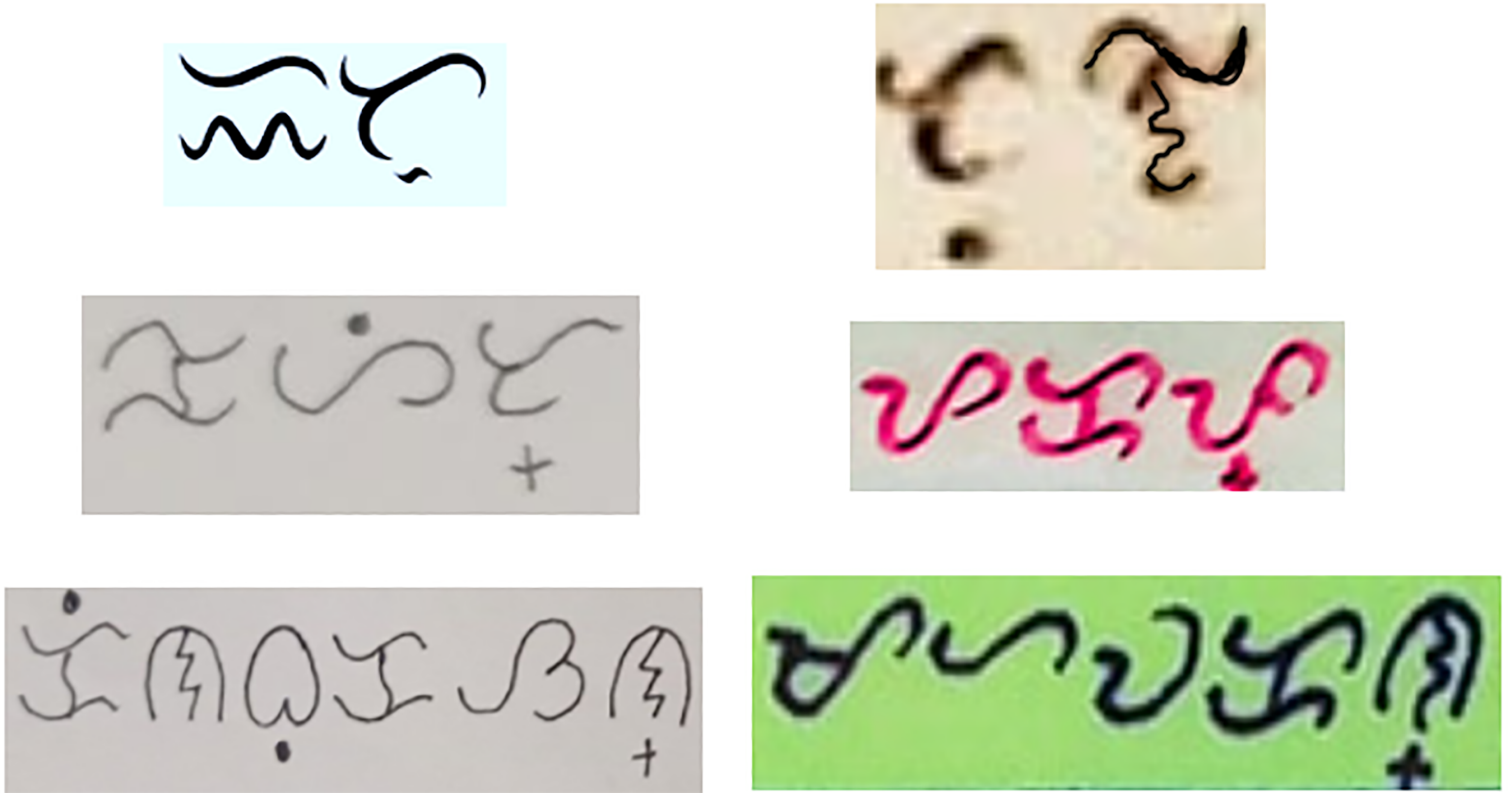
Some images of Tagalog words snipped from various websites.

Baybayin word images are taken from various websites. One thousand distinct Baybayin word images are saved with the use of a snipping tool. Some of the generated images are shown in Fig. 2.

**Figure 3:**
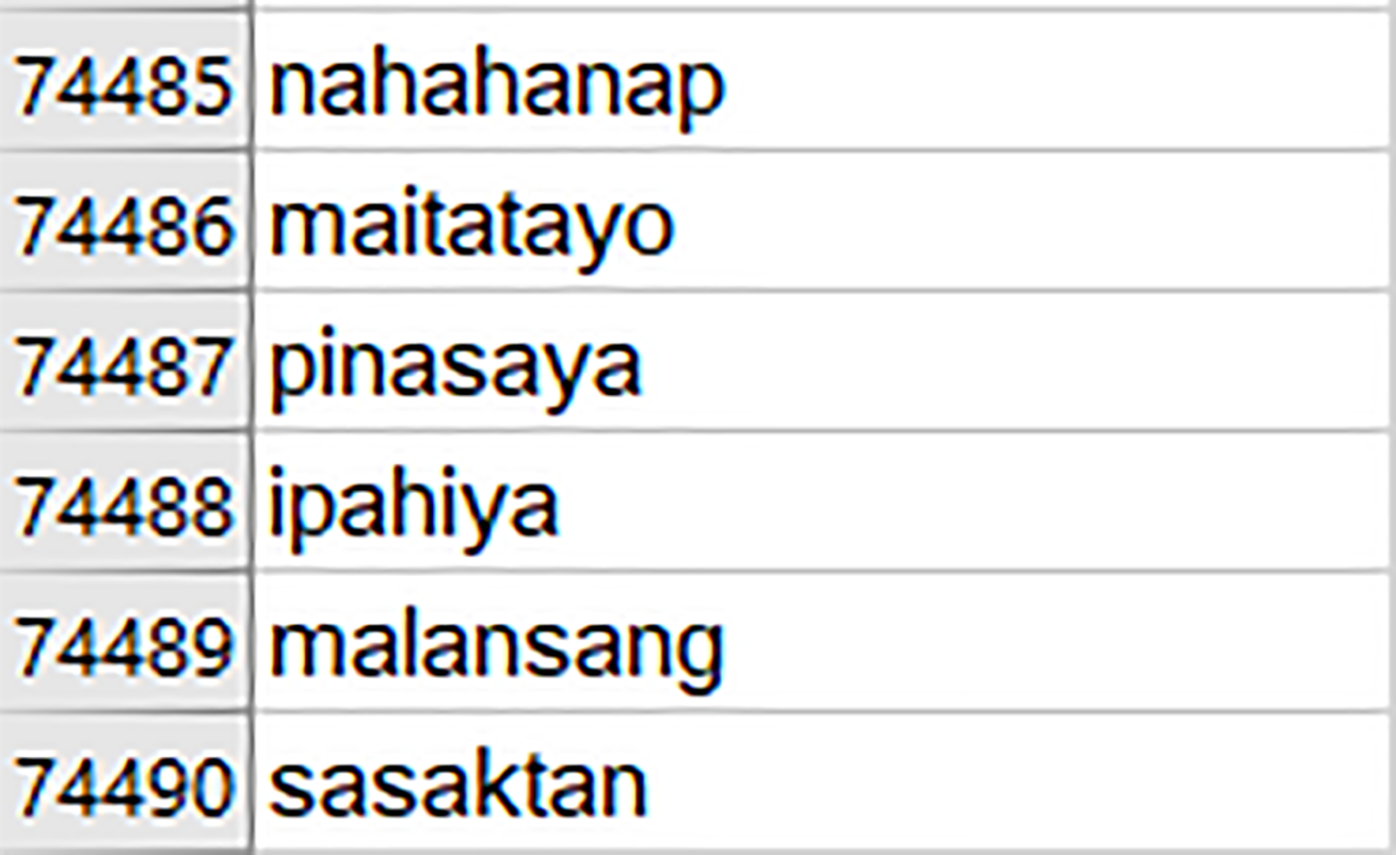
Sample entries of the Tagalog dictionary obtained from compiling 74,490 words from Tagalog word archives publicly available online.

Given an input image of a Baybayin word, the goal of the system is to identify its equivalent word/s written in Latin script. Because Baybayin does not differentiate ’e’ from ‘i’, ‘o’ from ‘u’, and ‘da’ from ‘ra’, the formed Latin script might not be a Tagalog word. Furthermore, a Baybayin word may have multiple transliterations. Examples of these occurrences are shown in Table 2. It can be seen in the first example that two different words with the same meaning are formed from the same Baybayin word. However, the second example illustrates that two words with different definitions can be found from the same Baybayin word. Thus, we need a database of Tagalog words to check all the possible equivalent words of a given image of a Baybayin word. In this work, we use a Tagalog dictionary that contains 74,490 Tagalog words. This dataset is obtained from publicly available Tagalog word archives on the internet. Figure 3 shows a preview of the said dictionary, which can be accessed through the repository (Pino, 2021b).

**Table 2:** Example of Baybayin words with multiple equivalent Latin translations. The words ’dinig’ and ’rinig’ have identical meanings in the Tagalog language. In English, both words mean *hear*. The words ’boto’ and ’buto’ are distinct Tagalog words that mean *vote* and *bone*, respectively.

Example	Baybayin word	Recognized latin equivalent	Other possible word conversion
1	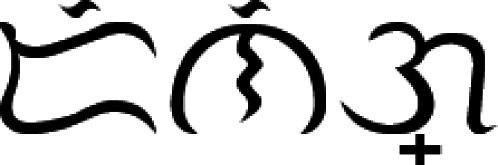	DINIG	RINIG
2	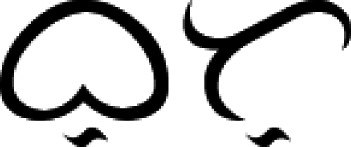	BOTO	BUTO

## Proposed System

The system presented here is coded and implemented using MATLAB (vR2020a). The proposed algorithm starts by identifying the characters in a Baybayin word using a segmentation algorithm. We use the SVM-OCR system proposed in Pino, Mendoza & Sambayan (2021) to categorize each character. The given input image must satisfy the following assumptions.

• The text print is darker than the background.
• The main body of the character is larger than its diacritic.
• The diacritic is not touching the main character, written above or below its respective main character, and is within the width of the main character.
• All Baybayin characters in the word are separated from each other.

The first three items above are assumptions of the SVM-OCR system to be used (Pino, Mendoza & Sambayan, 2021). The last assumption is to guarantee that the characters in the Baybayin word will be correctly extracted.

The classification process in the proposed algorithm relies on the two SVM classifiers generated in Pino, Mendoza & Sambayan (2021), namely, Baybayin characters classifier and the Baybayin diacritic classifier. SVM is one of the well-known classification algorithms in supervised machine learning. SVM starts with a set of training points/vectors ${\vec{x}}_{i}\in {\mathbb{R}}^{n},$i =1 , …, *N*, where *N* is the number of training points, and *n* is the number of features in a particular training sample. Each of these points belongs to one of two classes determined by a labeling variable *y*_*i*_ ∈ { − 1, 1}. In a (linearly) separable case problem, we can separate the two classes with a hyperplane, also known as the *linear classifier*, which can be written as

(1) \begin{eqnarray*}\vec{w}\cdot \vec{x}+b=0,\end{eqnarray*}

where $\vec{x}$, *b* and $\vec{w}$ are the input vectors, bias term, and weight vector, respectively. We want to maximize the separation distance of the two classes by creating two parallel lines so that no data points are between them. We produce these two parallel lines by fixing the functional margin from the hyperplane (Eq. (1)) to be equal to 1. Points that satisfy the conditions

(2) \begin{eqnarray*}\vec{w}\cdot \vec{x}+b\geq 1\end{eqnarray*}

and

(3) \begin{eqnarray*}\vec{w}\cdot \vec{x}+b\leq -1\end{eqnarray*}

are labeled 1 and −1, respectively. The region between the two hyperplanes represented in Eqs. (2) and (3) is called the *margin* and the distance between them is given by $ \frac{2}{\parallel \vec{w}\parallel } $. Thus, the objective is then given by solving the optimization problem

(4) \begin{eqnarray*}\begin{array}{@{}r@{}} \displaystyle & \text{minimize}_{\vec{w},b} & & \frac{1}{2} \parallel \vec{w}{\parallel }^{2} & \\ \displaystyle & \text{subject to} & & {y}_{i} \left( \vec{w}\cdot {\vec{x}}_{i}+b \right) \geq 1 \text{for all}i=1,\ldots ,N. \end{array}\end{eqnarray*}

The first-order optimality conditions of Problem (4) are determined using the Karush–Kuhn–Tucker (KKT) conditions. This is done by introducing Lagrange multipliers *α*_*i*_ on each term. Hence, optimal solutions ${\alpha }_{i}^{\ast }$, *b*^∗^, and ${\vec{w}}^{\ast }$ satisfy

\begin{eqnarray*}{\alpha }_{i}^{\ast } \left[ {y}_{i} \left( {\vec{w}}^{\ast }\cdot {\vec{x}}_{i}+{b}^{\ast } \right) -1 \right] =0. \end{eqnarray*}

This implies that if ${\vec{\alpha }}_{i}^{\ast }\not = 0$, then

\begin{eqnarray*}{y}_{i} \left( {\vec{w}}^{\ast }\cdot {\vec{x}}_{i}+{b}^{\ast } \right) =1, \end{eqnarray*}

where the data points ${\vec{x}}_{i}$’s determine the margins. These points are the *support vectors*. Let *S* be the set of indices of support vectors. Then, $\vec{x}\in {\mathbb{R}}^{n}$ can be categorized using

\begin{eqnarray*}f(\vec{x})=\text{sign} \left( \sum _{i\in S}{y}_{i}{\alpha }_{i}^{\ast } \left( {\vec{x}}_{i}\cdot \vec{x} \right) +{b}^{\ast } \right) . \end{eqnarray*}

The entire formulation can be applied to the nonseparable case problem (nonlinear). Boser, Guyon & Vapnik (1992) proposed that the each data point $\vec{x}$ in the input space is mapped to a point $\phi (\vec{x})$ in a higher dimensional space, called the *feature space*, where a separating hyperplane can be found. With the aid of Mercer’s theorem, the construction of the linear classifier is possible if $\phi (\vec{{x}_{i}})\cdot \phi (\vec{{x}_{j}})$ can be written as a kernel function $\kappa (\vec{{x}_{i}},\vec{{x}_{j}})$ for any *x*_*i*_, *x*_*j*_ ∈ ℝ^*n*^. This technique is known as the *kernel trick* and the decision function now has the form:

(5) \begin{eqnarray*}f(\vec{x})=\text{sign} \left( \sum _{i\in S}{y}_{i}{\alpha }_{i}^{\ast }\kappa \left( {\vec{x}}_{i},{\vec{x}}_{j} \right) +{b}^{\ast } \right) .\end{eqnarray*}

Notice in (5) that the function is not dependent on the dimensionality of the feature space. The Radial Basis Function (RBF) kernel functions have been used in experiments presented in Pino, Mendoza & Sambayan (2021) as it has shown its effectiveness than other kernel functions in classifying script characters (Sok & Taing, 2014; Tautu & Leon, 2012).

**Figure 4:**
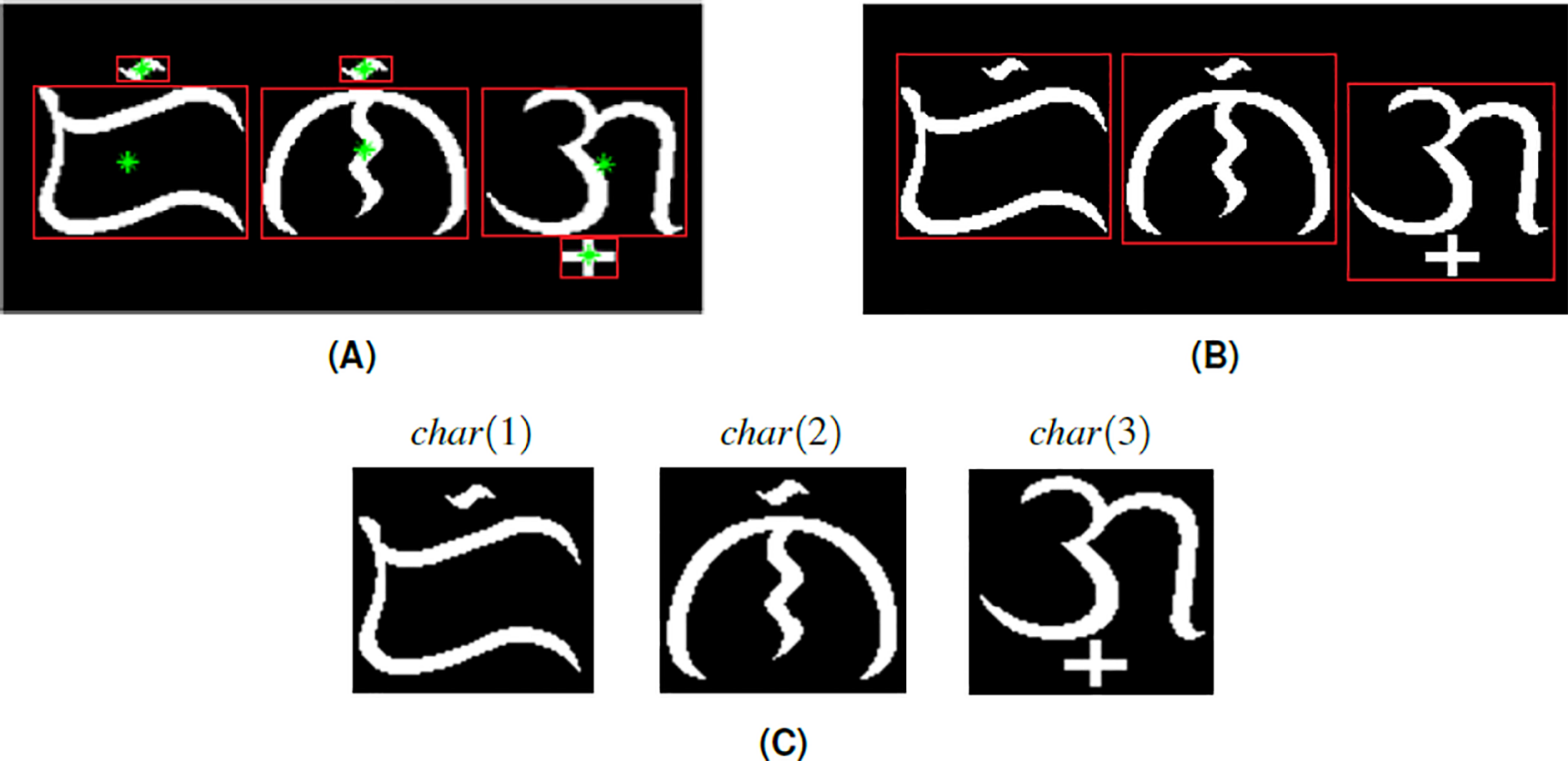
Segmenting an image of a Baybayin word into its character components: (A) bounding box from ocr function with each component’s centroid superimposed, (B) computed bounding box for each character, (C) the segmented character components *char*(*k*).

To carry on with the proposed system, the input image of a Baybayin word is first converted to binary data using a modified *k* − means function. Then, we implement the MATLAB built-in ocr function and acquire the text properties: bounding box, area, and centroids. Using the computed bounding boxes, we perform a segmentation method. This operation allows us to separate each character from the binary image. A modification has to be made on the segmentation method because the result provided by ocr also assumes the accents as separate components. For instance, in Fig. 4A, the ocr function returns 6 character locations - three main body components and three diacritics. The modification is done in two steps:

1. If the absolute difference between values of the *x* −coordinates of the centroids of two components is within a given threshold, the system treats the two components as one. To illustrate, the centroids of the main body and accent are shown in Fig. 4A (green dots). One can see that the centroids are nearly aligned.
2. The bounding box of the combined characters is recomputed based on the bounding boxes of the components that are part of the main character identified in step 1 (see Fig. 4B).

**Table 3:** Finding other possible translations of an image of a Baybayin word. The second column shows a recognized Latin equivalent word obtained by the system. The third column displays all the other possible words constructed given the input image. The italicized word in bold is a word found in the Tagalog dictionary. The words *itodo* and *ituro* are distinct Tagalog words which mean *to go all out* and *to teach*, respectively. When the image in the first column is plugged into the proposed system, the outputs are *itodo* and *ituro*.

Baybayin Word	Recognized latin equivalent	New strings from alteration of syllables		
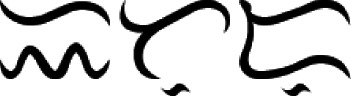	itodo	• etodo	• itudo	• eturu
		• etodu	• itudu	• itoro
		• etudo	• etoro	• itoru
		• etudu	• etoru	•***ituro***
		• itodu	• eturo	• ituru

**Figure 5:**
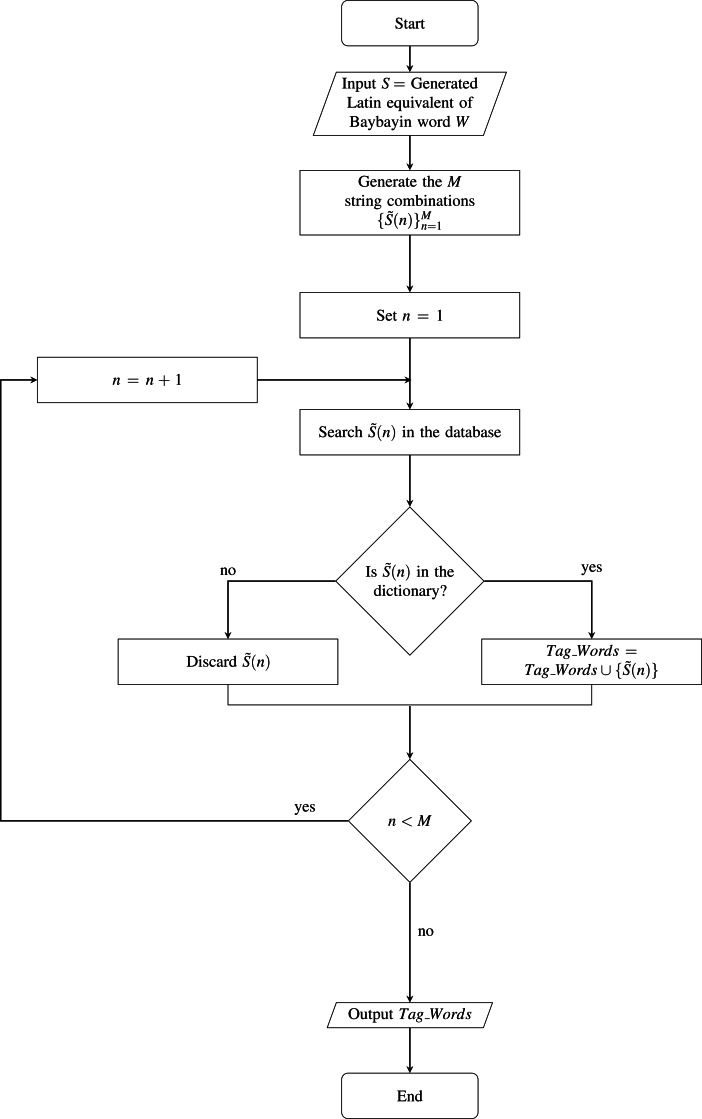
Flowchart of Extra Tagalog Word Finder.


 
We define *W* as the set of characters ${\{char(k)\}}_{k=1}^{N},$ where *N* denotes the number of Baybayin characters in the word (see Fig. 4C). Each character *char*(*k*) in *W* is converted to Latin script. The resulting *N* syllables are concatenated to form the word *S*, then cross-checked in the Tagalog dictionary. If *S* is in the dictionary, the word is included in the set of possible Latin transliterations, *Tag*_*Words*. Then, it is checked if any of ‘e/i’, ‘o/u’, or ‘d/r’ appears in *S*. If so, we look for other possible Tagalog words by checking all the combinations. An example of this process is shown in Table 3. In this example, 16 words can be constructed from a single Baybayin word. Among these, only two words are found in the Tagalog dictionary. To find all the Tagalog words, Algorithm 1 is performed. This operation involves changing syllables of *S* that don’t have a unique representation. This alteration process is combined with the other syllables to form a new string that could potentially be a Tagalog word. Each formed string is cross-checked in the Tagalog dictionary. All strings found in the dictionary are added to the set *Tag*_*Words*. The flow of this process is illustrated in Fig. 5.

______________________________________________________________ 
Algorithm 1 Extra Tagalog Word Finder 
______________________________________________________________ 
Require: S = Generated Latin equivalent of Baybayin word W 
Ensure: Potential Tagalog words found. 
  1:  Generate the M string combinations { ˜S(n)}Mn=1. 
  2:  Set n = 1. 
  3:  for n = 1 : M do 
 4:     Search  ˜ S(n) in database. 
  5:     if  ˜ S(n) is the dictionary then 
 6:         Tag_Words = Tag_Words ∪{ ˜S(n)}. 
  7:     else 
 8:         Discard  ˜ S(n). 
  9:     end if 
10:  end for 
11:  Tag_Words = the set of all possible Tagalog words found. 
________________________________________________________________ 
 
    


**Figure 6:**
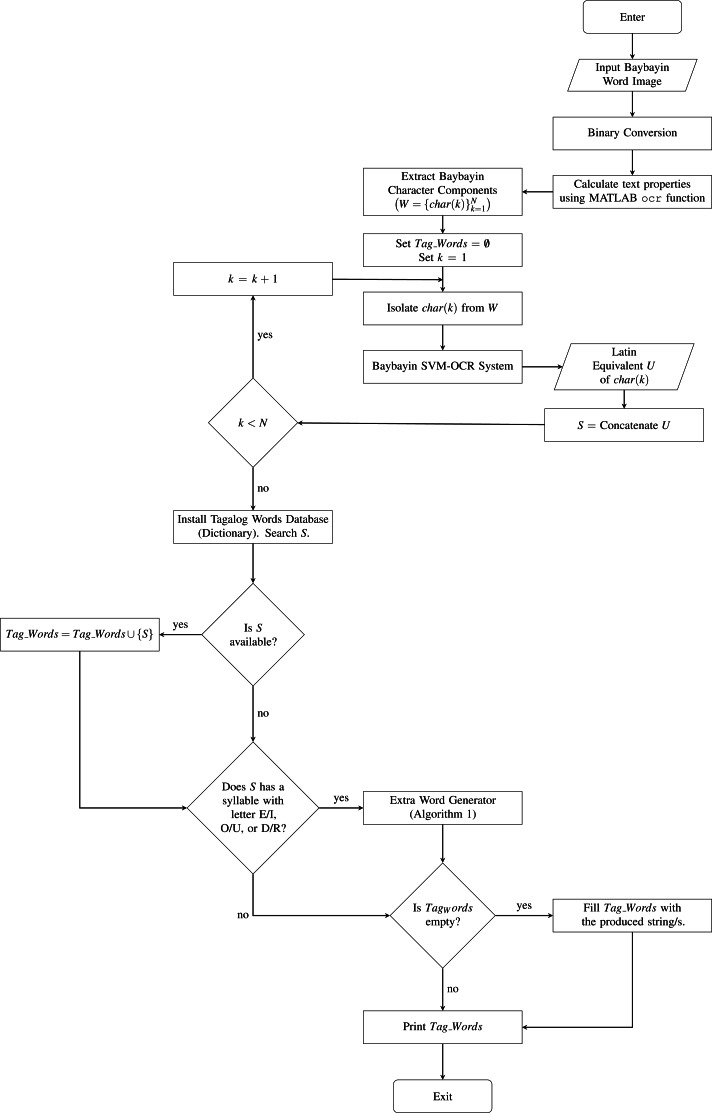
The Proposed System.


 
After finding all the extra words, the system prints out *Tag*_*Words*. The proposed system is summarized in Fig. 6 and Algorithm 2. Although the collection of words in the Tagalog dictionary is already composed of 74,490 words, the database is not exhaustive. Thus, it is still possible that all the generated strings for a Baybayin word are not in the dictionary. This can happen if the Baybayin in the image represents a proper noun, a name, or a foreign word. In this case, the system will tell the user that the word is not in the dictionary and will display all the strings.

__________________________________________________________________________________ 
Algorithm 2 The Proposed System 
__________________________________________________________________________________ 
Require: Image containing a Baybayin word 
Ensure: All possible Latin conversion of word W. 
  1:  Binary conversion of the input image data. 
  2:  Compute the text properties using MATLAB ocr function. 
  3:  Extract each character components to form W = {char(k)}Nk=1. 
  4:  Set Tag_Words = ∅. Set k = 1. 
  5:  for k = 1 : N do 
 6:     Feed char(k) into Baybayin Character OCR System. 
  7:     U = Equivalent output unit. 
  8:     S = concatenate U. 
  9:  end for 
10:  Load the Tagalog Dictionary and search S in it. 
11:  if S is available then 
12:     Tag_Words = Tag_Words ∪{S}. 
13:     if S has a syllable that incorporates vowel E/I or O/U, or a letter D then 
14:         Implement Algorithm 1 
15:     end if 
16:     Tag_Words = the set of all possible equivalent words in Latin script of 
        W. 
17:  else 
18:     if S has a syllable that incorporates vowel E/I or O/U, or a letter D then 
19:         Implement Algorithm 1. 
20:         if Tag_Words = ∅ then 
21:            Tag_Words = Tag_Words ∪{S}∪⋃m 
   n=1{ ˜S(n)}. 
22:         end if 
23:     else 
24:         Tag_Words = Tag_Words ∪{S}. 
25:     end if 
26:     Tag_Words = the set of all possible equivalent words in Latin script of 
        W. 
27:  end if 
____________________________________________________________________________________    


## Recognition Setup, Results and Discussions

**Figure 7:**
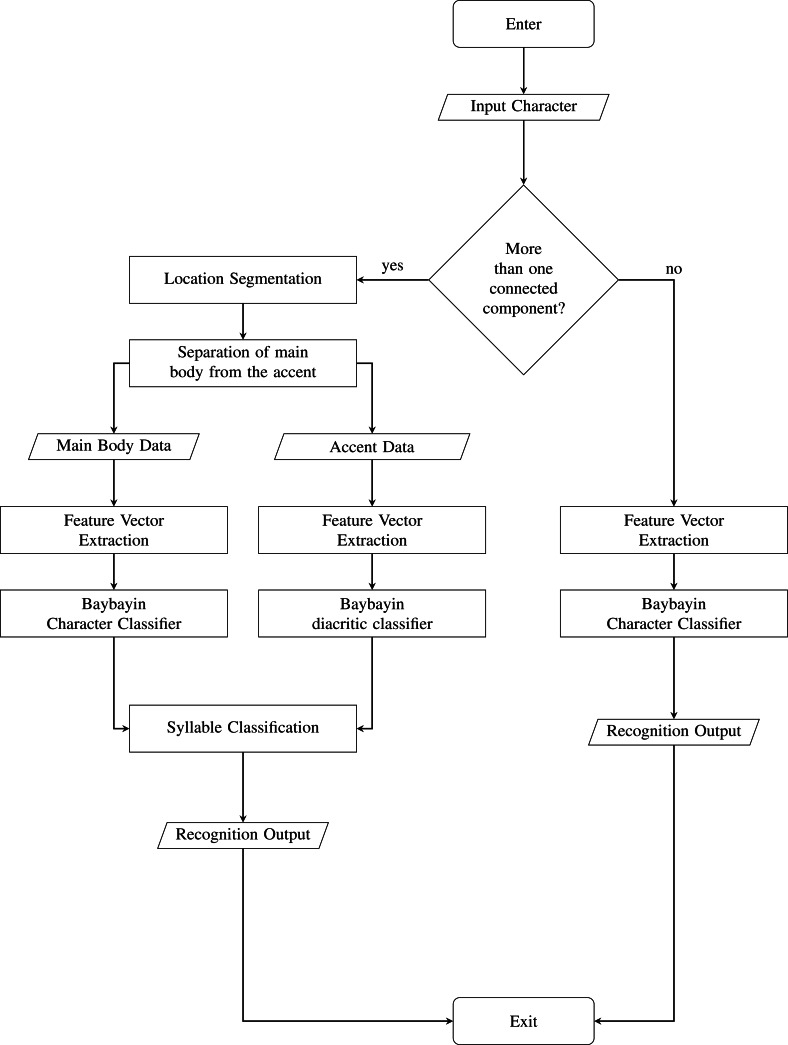
Baybayin Character OCR System.

We test the proposed system to 1000 images of Baybayin words publicly available in Pino, (2021a). To the best of our knowledge, this is the first dataset provided for Baybayin word images. These images satisfy the system’s assumptions stated in ‘Proposed System’. The SVM Baybayin character model and the SVM Baybayin diacritic classifier utilized in Pino, Mendoza & Sambayan (2021) are used for classifying each character in the input Baybayin word. Both models have classification rates of more than 96%. We modified the system presented in Pino, Mendoza & Sambayan (2021), where its focus is on identifying Baybayin characters only. Figure 7 shows the modified system. Similar feature extraction techniques are then carried out to process and classify the Baybayin character. Its output is the Latin syllabic equivalent of the Baybayin character. For instance, when the character *char*(1) in Fig. 4C is fed to the SVM-OCR system, its potential output is ‘de’ or ‘di’ (see Fig. 1). The same method applies to the rest of the *char*(*k*)’s and then orderly concatenated to generate the corresponding word *S*.

**Figure 8:**
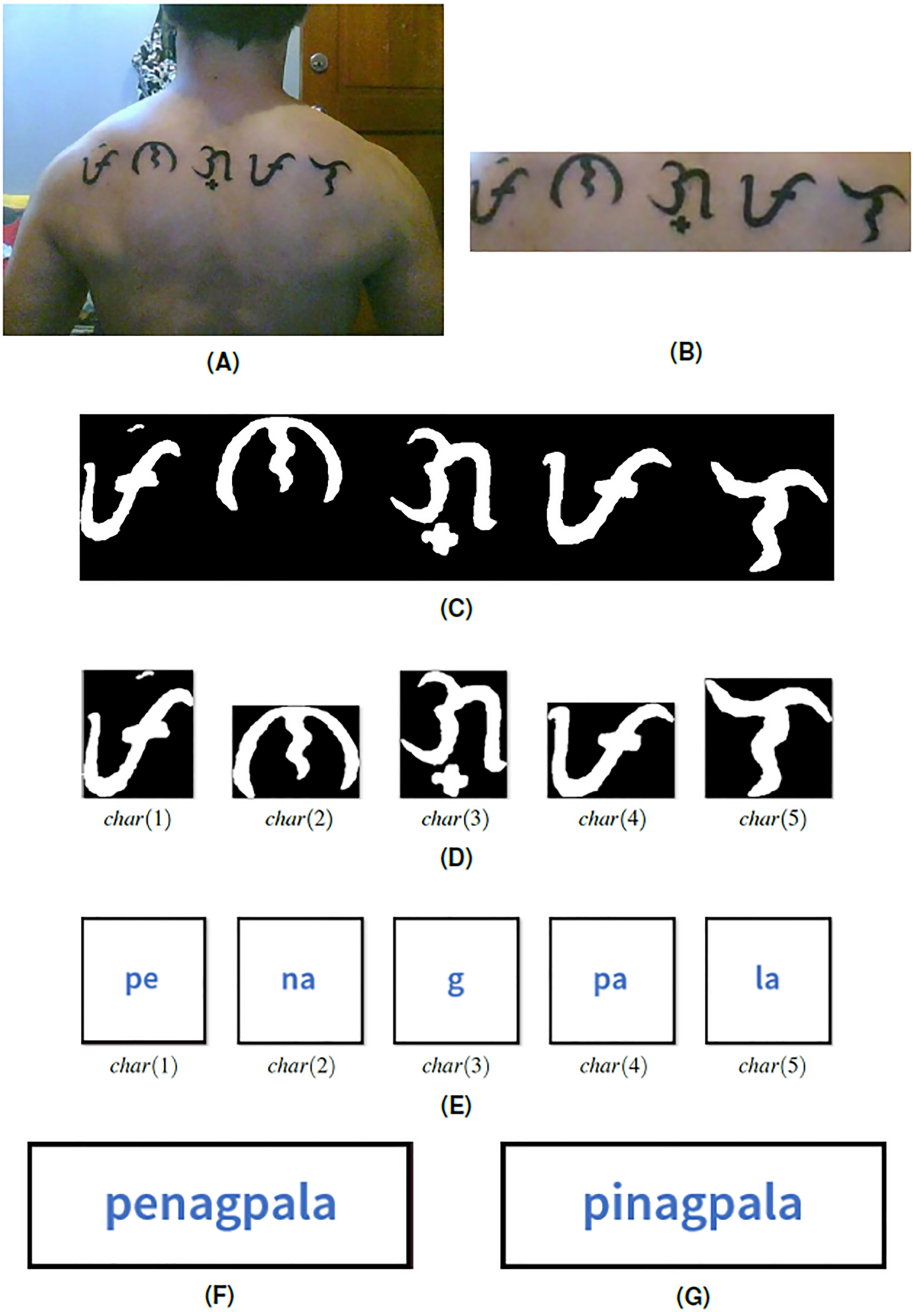
(A) Baybayin tattoo, (B) cropped Baybayin word, (C) binarized image, (D) word-to-character segmentation, (E) Baybayin character SVM-OCR system output, (F) a generated equivalent word written in Latin alphabet, and (G) a Tagalog word found in the dictionary using Algorithm 1.

 A test is successful if the equivalent word is found in the Tagalog dictionary. Thus, a misclassification of at least one character could prompt a recognition error. The provided MATLAB script will display the following text to indicate that the generated word is not in the dictionary:

‘ The word is not in the dictionary. The possible translations are as follows...’

After implementing the proposed system to the dataset, 979 Baybayin word images were correctly transliterated. This interprets to a 97.9% recognition accuracy, which is computed using the formula

\begin{eqnarray*}\text{recognition accuracy}= \frac{\text{number of correctly transliterated words}}{\text{total number of test words}} \times 100\text{%}. \end{eqnarray*}

To illustrate the whole process, we apply our proposed system to an image containing a Baybayin tattoo (Fig. 8A). The Baybayin word is cropped from the image before feeding it to the system (Fig. 8B). The algorithm begins with converting the input image to binary data (Fig. 8C). Then, the system implements the word-to-character segmentation process where it detects five Baybayin characters (Fig. 8D). The procedure is followed by feeding each *char*(*k*) to the Baybayin SVM-OCR system and obtaining their corresponding Latin equivalent words (Fig. 8E). Each character recognition result is then concatenated orderly to form the equivalent word in Latin script (Fig. 8F). Using Algorithm 1 and the Tagalog dictionary, the system obtained the Tagalog word ‘pinagpala’, which means *blessed* in the English language.

**Figure 9:**
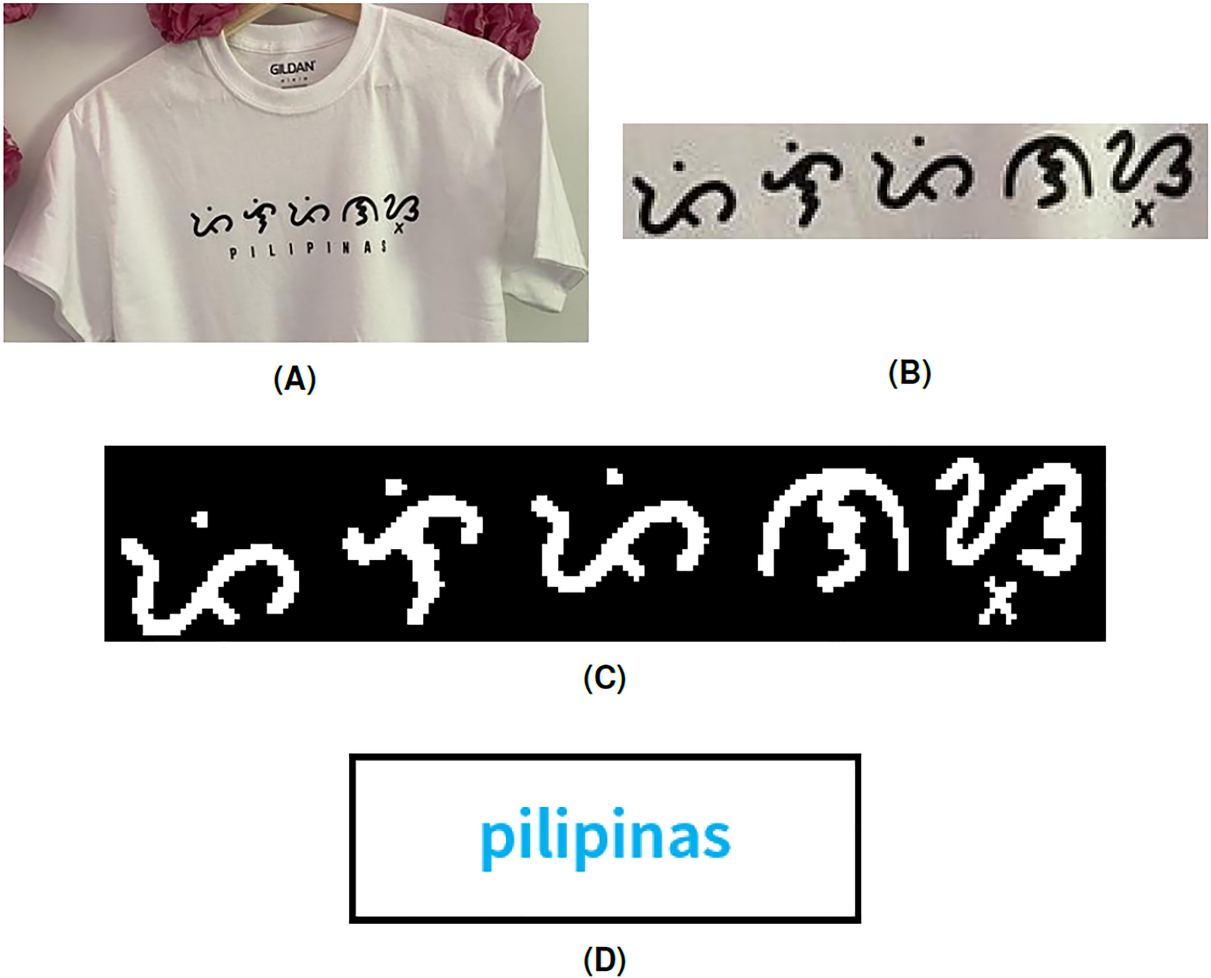
(A) Baybayin themed shirt, (B) cropped Baybayin word, (C) binarized image, and (D) a generated equivalent word written in Latin alphabet.

Another sample simulation is implemented to identify the Latin equivalent of a Baybayin print on a T-shirt. ’Pilipinas’ is the Tagalog word for the *Philippines*. Again, the system correctly translated the Baybayin word as shown in Fig. 9.

**Figure 10:**
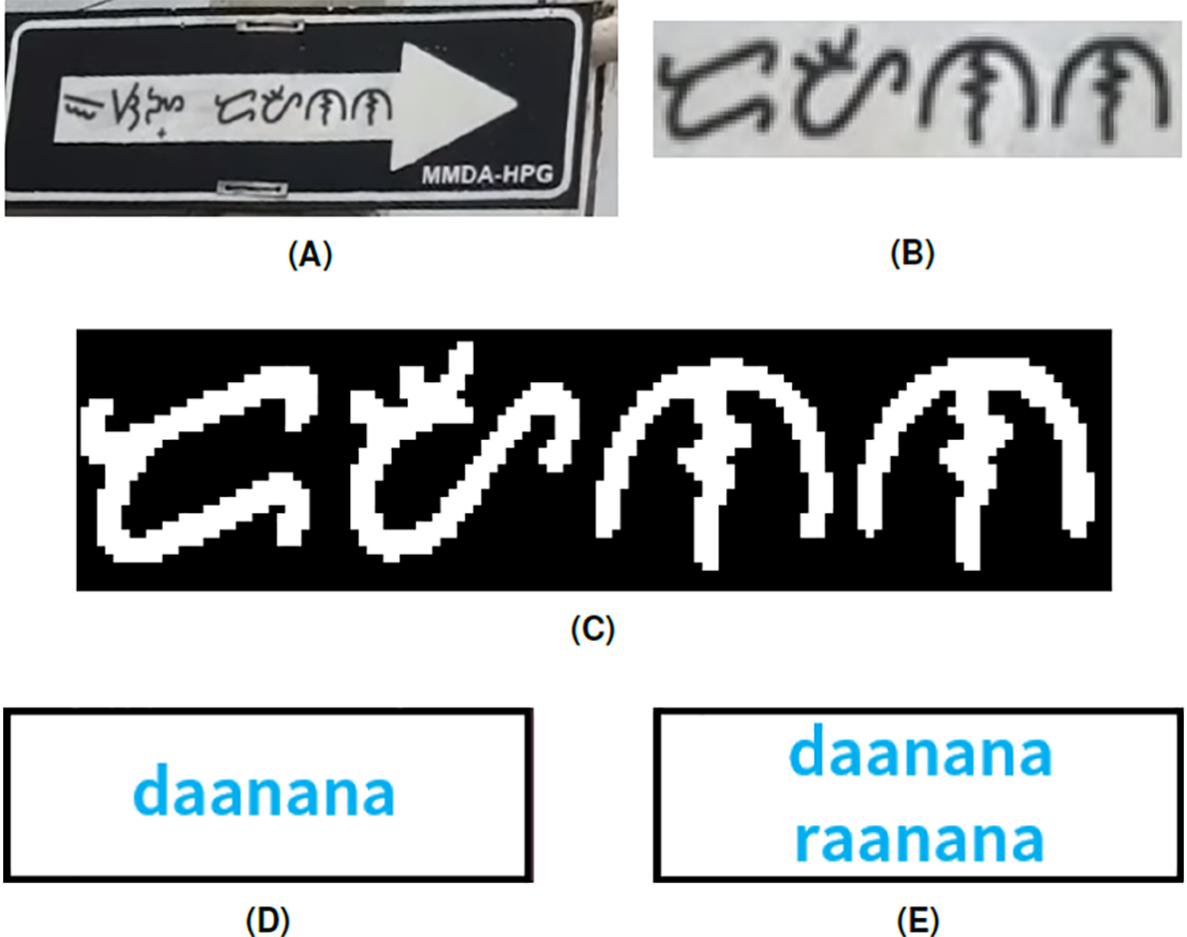
(A) Baybayin signage, (B) cropped Baybayin word, (C) binarized image, and (D) generated equivalent word written in Latin alphabet. Since the word is misspelled, the word was not found in the dictionary. Hence, the system generated all the possible word combinations based on the diacritics or characters with multiple transliterations (E).

The example in Fig. 10 shows the conversion of a Baybayin word on a signage into Latin. The second word in the signage (Fig. 10A) is not included in the Tagalog dictionary because the last character in the Baybayin word is missing a diacritic. Thus, the algorithm will tell the user that the word is not in the database and display all the possible conversions. In this case, the possible strings are ‘daanana’ and ‘raanana’. These are incorrect spellings of ‘daanan’, which means *way*. Our proposed system does not recognize misspelled Baybayin words. Another similar scenario is when the Baybayin word pertains to a proper noun (e.g., name of a person), which might not be included in the dictionary. To resolve this, one can expand the database of the dictionary to include proper nouns and other relevant words.

**Figure 11:**
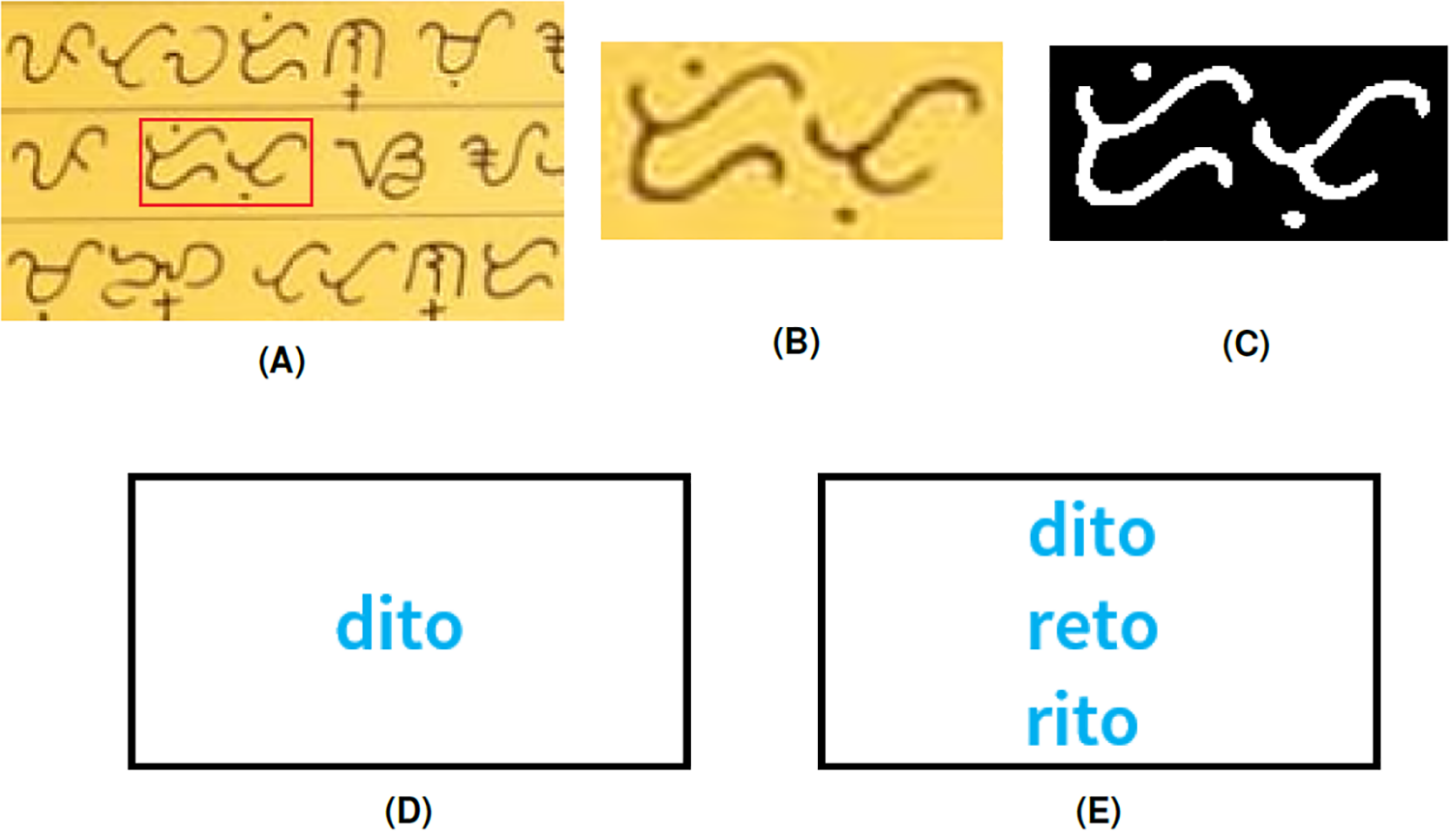
(A) Baybayin texts, (B) cropped Baybayin word, (C) binarized image, (D) generated equivalent word written in Latin alphabet, and (E) all the other equivalent Tagalog words found in the dictionary using Algorithm 1.

The last example in Fig. 11 shows how the algorithm can identify multiple translations of one Baybayin word. The Baybayin word is equivalent to three Tagalog words in the database. The Tagalog words ’dito’ and ’rito’ both mean *here*. The Tagalog word ‘reto’ means *to introduce someone to another person as matchmaking*.

These simulations illustrate how our proposed system can be used in transliterating Baybayin texts transcribed in old books, tattoos, graphic designs, signage, and documents, among others. Baybayin was commonly used in the 1500s. Hence, a lot of historical documents during the pre-Hispanic times are written in Baybayin. Our system can help researchers read the Baybayin words written in these old documents.

## Conclusions and Future Works

Several machine learning algorithms have been studied in identifying Baybayin characters. However, none has been done in identifying Baybayin at the word level. The main contribution of this paper is to propose a system for Baybayin word recognition, where we determine all corresponding Latin transliteration. To the best of our knowledge, the proposed system is the first of its kind for recognizing Baybayin scripts at word level. The system relies heavily on previous work on Baybayin character recognition Pino, Mendoza & Sambayan (2021). The method is tested on a novel dataset found in Pino (2021a), where it contains 1000 Baybayin word images and yielded a competitive recognition accuracy of 97.9%.

The system was conceived under certain assumptions. Although these assumptions are not restrictive, it will be interesting to know how the system can be modified for more general use. The datasets for the Baybayin images and Tagalog dictionary can also be expanded.

Baybayin is written depending on how the word is pronounced. Thus, a system for recognizing proper nouns, names, or foreign words will be tricky. A possible approach to resolve this is by first converting a given Baybayin word into its equivalent international phonetic alphabet transliteration before identifying the equivalent Latin script. This is an exciting research direction. One can also explore how the system can perform in identifying Baybayin words or phrases in a document. This will not be trivial because of the multiple transliterations of a word written in Baybayin. Identifying the correct word from various choices requires delving into the syntax of the Tagalog language. Nevertheless, this an interesting topic to look into. Another research direction is identifying misspelled Baybayin words just like in the example shown in Fig. 10. A mobile application based on our proposed system can also be developed.

We hope that this work will help promote Baybayin and encourage researchers to pursue studies on the computer vision of Baybayin. We strongly recommend that other word-level recognition schemes for Baybayin scripts be studied. Perhaps, alternative machine learning algorithms can be used. Once these other methods are explored, a comparative study can be done.

## Additional Information and Declarations

### Data Availability

The following information was supplied regarding data availability: The dataset for the images containing Baybayin words are available at Kaggle: Rodney Pino, “Baybayin Word Images.” Kaggle, 2021, doi: 10.34740/KAGGLE/DSV/1977618. The MATLAB source codes and the database of Tagalog dictionary used in this study are available at GitHub: https://github.com/rbp0803/A-Baybayin-Word-Recognition-System.
